# Artificial Intelligence in Triple-Negative Breast Cancer: Applications in Diagnosis, Treatment Response, and Prognosis

**DOI:** 10.3390/diagnostics16050671

**Published:** 2026-02-26

**Authors:** Ziyu Fu, Xiaofei Huo, Andrew B. Jing, Jingfei Ma, Gaiane M. Rauch

**Affiliations:** 1 Department of Imaging Physics, The University of Texas MD Anderson Cancer Center, Houston, TX 77030, USA; 2 Department of Abdominal Imaging, The University of Texas MD Anderson Cancer Center, Houston, TX 77030, USA

**Keywords:** triple negative breast cancer, artificial intelligence, MR imaging, diagnosis, staging, neoadjuvant therapy

## Abstract

Triple-negative breast cancer (TNBC) is an aggressive breast cancer subtype associated with limited targeted treatment options, heterogeneous treatment response, and high risk of early recurrence. Artificial intelligence (AI) has rapidly emerged as a powerful tool to address key clinical challenges in TNBC across diagnosis, treatment response assessment, and prognosis. Diagnostic and staging challenges persist due to variable imaging features in TNBC and limitations in conventional modalities, increasing the risk of delayed detection. Predicting response to neoadjuvant systemic therapy remains difficult, as patient responses are heterogeneous, and existing clinical markers provide limited early predictive value. Prognostication in TNBC is similarly constrained by the absence of widely used genomic tools and reliance on clinicopathologic factors that incompletely reflect tumor biology. This review summarizes recent advances in AI applications for TNBC across diagnosis, tumor characterization and staging, treatment response prediction, and prognosis, highlighting both emerging opportunities and current limitations in clinical translation.

## 1. Introduction

Triple-negative breast cancer (TNBC) is an aggressive and clinically challenging subtype of breast cancer, defined by the absence of estrogen receptor, progesterone receptor, and HER2 expression. TNBC accounts for approximately 20% of all breast cancers and is associated with higher rates of early recurrence, visceral metastasis, and cancer-specific mortality compared with hormone receptor–positive or HER2-positive disease [1,2]. Because TNBC lacks established therapeutic targets, systemic treatment for locally advanced diseases has historically relied on cytotoxic chemotherapy, with immunotherapy more recently incorporated. Despite therapeutic advances, outcomes remain heterogeneous, reflecting substantial interpatient variability in tumor biology and treatment response. These features make TNBC an area of unmet clinical need across diagnosis, treatment response, and prognosis.

Accurate diagnosis and staging of TNBC is challenging. Conventional breast imaging modalities, including mammography and ultrasound, often demonstrate limited sensitivity for TNBC [3]. Breast MRI offers high sensitivity; however, it is limited by cost, access, and relatively high false-positive rates. Precise staging, including assessment of tumor extent and lymph node involvement, is critical for treatment planning but remains difficult using standard imaging alone. Artificial intelligence (AI) offers a compelling opportunity to address these challenges by improving the extraction and interpretation of complex imaging information. Radiomics-based approaches quantify imaging features that are not visually apparent, and deep-learning methods can learn implicit patterns directly from raw imaging data. These approaches are well-suited to TNBC image interpretation, where subtle radiologic differences may reflect underlying biological heterogeneity that is difficult for human observers to recognize.

Beyond diagnosis, predicting treatment response represents another major clinical challenge in TNBC. Neoadjuvant systemic therapy (NAST) is now the standard of care for most patients, including those with early-stage disease, as well as those with locally advanced cancer. Given the toxicity of multi-agent chemotherapy and immunotherapy regimens, the ability to identify likely responders from non-responders is especially important in TNBC. AI-based models, particularly deep learning applied to multiparametric and longitudinal imaging, are well-positioned to address this need. By integrating information from multiple MRI sequences and time points, AI systems can capture dynamic changes in tumor vascularity, cellularity, and morphology that may precede visible size reduction. In TNBC, where therapeutic windows are narrow and early disease progression can be rapid, the potential clinical value of early response prediction can be significant.

Long-term prognosis and risk stratification constitute a third critical area where TNBC presents unique challenges. TNBC is characterized by a higher risk of early recurrence, particularly within the first three to five years after diagnosis, and by a greater propensity for visceral and central nervous system metastases compared with other subtypes [4,5]. Unlike hormone receptor–positive breast cancer, there are no widely adopted genomic assays routinely used to guide prognostication or adjuvant treatment decisions in TNBC. As a result, risk assessment relies largely on clinicopathologic factors that may not fully capture underlying tumor aggressiveness. AI-based prognostic modeling seeks to fill this gap by leveraging imaging, pathology, and clinical data to improve the prediction of recurrence and survival. These approaches offer the advantage of using routinely acquired data and may provide noninvasive or scalable alternatives to molecular testing.

**Figure 1:**
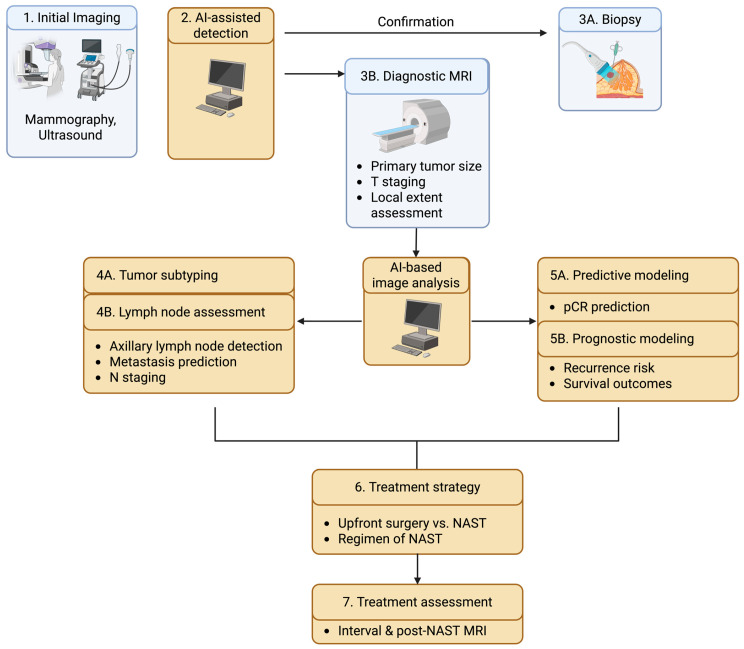
Potential clinical workflow of triple-negative breast cancer with AI integration. (NAST = neoadjuvant systemic therapy; pCR = pathological complete response).

The following sections provide a narrative overview of recent advances in AI applications across the clinical management of TNBC. Rather than cataloging all published work, we focus on representative studies that illustrate emerging methodological directions, common design strategies, and practical considerations for clinical translation. We first examine AI-assisted approaches for diagnosis, tumor characterization, and staging, followed by a section reviewing AI models developed to predict response to neoadjuvant therapy, focusing on the nuanced considerations in incorporating multiparametric and longitudinal imaging. Finally, we discuss AI-based methods for prognosis, recurrence prediction, and risk stratification. We aim to highlight both the promise and the limitations of current approaches. Together, these developments underscore the growing role of AI as an adjunctive tool to address the unique clinical challenges posed by TNBC. A potential AI-integrated workflow for clinical management of TNBC is summarized in Figure 1.

## 2. AI for Diagnosis and Tumor Characterization

### 2.1. Current State of TNBC Diagnosis

Existing approaches for diagnosing TNBC include mammography, ultrasound, and possibly MRI. Each of the approaches has certain limitations. Mammography, for instance, frequently results in false-negative findings or delayed diagnosis because TNBC often lacks characteristic calcifications or distinctive radiographic features, a problem that is exacerbated in women with dense breast tissue [6]. Breast ultrasound is also limited, as its accuracy is highly dependent on operator experience. Retrospective analysis is challenging, and differentiation between benign and malignant lesions remains difficult [6,7].

Although MRI offers high sensitivity, its clinical utility is restricted by a substantial rate of false-positive results. Together, these limitations underscore the urgent need for more accurate and efficient diagnostic strategies. In this context, AI has emerged as a promising solution, with the potential to improve diagnostic accuracy and minimize subjectivity [8].

### 2.2. AI for TNBC Subtypes

Over the past decade, a growing hypothesis has emerged that AI-based quantitative analysis of breast imaging can reveal subtle tumor characteristics linked to specific receptor subtypes of breast cancer that are not discernible to the human eye. This concept has been substantiated by multiple AI studies demonstrating moderate to high diagnostic performance in TNBC across different MRI modalities, with reported AUC values ranging from 0.73 to 0.96 in the last 5 years (Table 1). In particular, the integration of AI with multiparametric MRI (MP-MRI) radiomics has shown strong potential for differentiation of TNBC from other breast cancer subtypes [7,8]. Leithner et al. investigated the effectiveness of radiomics with AI tools applied to MP-MRI for identifying receptor subtypes of breast cancer [9]. Notably, this was one of the first studies to assess combined dynamic contrast-enhanced MRI (DCE-MRI) and diffusion-weighted imaging (DWI) radiomic signatures using a strictly standardized imaging protocol and an advanced neural network model. Their findings revealed a median AUC of 0.86 when a multilayer perceptron feed-forward artificial neural network was used to distinguish TNBC from all other breast cancers. Similarly, Huang et al. employed an MP-MRI–based radiomics approach in a cohort of 188 breast cancer patients and demonstrated that a machine-learning classifier could identify TNBC with an AUC of 0.86 [10]. Consistent results were reported in another study, which showed that selected radiomic features extracted from MP-MRI were good predictors of breast cancer subtypes. In that work, six classification algorithms were evaluated, with the DL classifier achieving the highest diagnostic performance for differentiating TNBC from non-TNBC cases in the testing set, with an AUC of 0.965 and an accuracy of 0.926 [11]. Comparable findings were also reported by Zhang et al. in a larger cohort of 477 patients, where radiomics derived from MP-MRI achieved strong classification performance [12]. Their study additionally showed that classifiers trained on MP-MRI data (AUC = 0.75–0.90) significantly outperformed those based on individual MRI sequences alone (AUC = 0.62–0.88). On the other hand, Romeo et al. demonstrated that ML-driven radiomics models using 18F-FDG PET/MRI could accurately discriminate TNBC from other breast cancer subtypes [13].

Beyond breast MRI, numerous investigations have explored the application of AI to breast ultrasound and mammography for differentiating TNBC from other subtypes [21,22,23,24,25,26,27]. Advanced ML models, such as logistic regression and classification algorithms, have been applied to ultrasound imaging, incorporating both grayscale (GS) and color Doppler (CD) features. Wu et al. assessed the diagnostic value of GS and CD ultrasound features extracted via ML for distinguishing TNBC from non-TNBC, reporting AUCs of 0.85 and 0.65, respectively [28]. When GS and CD features were combined, AUC further improved to 0.88. This performance notably surpassed that of conventional visual image interpretation, suggesting that such AI-assisted approaches may meaningfully support clinical decision-making in the future. Similarly, Boulenger et al. developed a deep-learning model to automatically differentiate TNBC from other subtypes using only ultrasound images and clinical data, achieving an AUC of 0.86 [29]. With mammography, computerized detection techniques have shown marked improvements in lesion identification and diagnostic accuracy compared with traditional assessment, particularly in patients with dense breast tissue, with a reported TNBC detection rate of approximately 75% [30]. Ma et al. further contributed by developing interpretable ML models to differentiate TNBC subtypes using combined clinical and imaging indicators [31]. Among five evaluated models, the decision tree (DT) approach demonstrated the best performance in distinguishing TNBC from other breast cancer variants, achieving an AUC of 0.971 and an accuracy of 0.947.

Across different breast imaging modalities, radiomics combined with ML classifiers has emerged as the most widely adopted AI strategy for TNBC differentiation. However, in another study by Ma et al., radiomic features extracted from mammography were used to identify TNBC, yielding an AUC of 0.865, which was lower than that achieved by the DT model [21]. This reduced performance may be attributed to the susceptibility of radiomics to variations in image preprocessing and feature extraction, as well as its reliance on single-modality imaging data [31]. The study also compared diagnostic performance among four radiologists in distinguishing TNBC from other breast cancers, both with and without DT model assistance. Notably, the inclusion of the DT model led to improvements in accuracy, sensitivity, and specificity for the radiologists, underscoring the potential of interpretable AI tools to enhance clinical diagnostic performance.

### 2.3. AI for Staging

MRI findings often play a significant role in determining whether patients should undergo upfront surgery or receive neoadjuvant chemotherapy, especially when assessing the tumor size and clinical T stage [32]. Although AI models have been extensively applied for tumor detection, few studies have explored their diagnostic potential in the context of tumor size estimation and clinical staging. Conte et al. developed a radiomics-based AI approach using DCE-MRI to differentiate in situ from invasive breast cancer, achieving the highest AUC of 0.81 [33]. Other similar studies were conducted with various AUCs from 0.70 to 0.90 [34,35,36,37,38,39]. Park et al. demonstrated substantial agreement between MRI and AI-guided mammographic T staging, which may help to offer supportive information in situations where MRI is unavailable or limited [40].

Accurate identification of lymph node metastasis is crucial for staging. Preoperative assessment of nodal involvement provides important guidance for surgical planning and treatment strategies [41]. In patients with early-stage breast cancer and clinically negative lymph nodes, sentinel lymph node biopsy detects axillary lymph node metastasis in approximately 15–20% of the patients [42]. Small axillary lymph nodes containing micrometastases cannot be detected by conventional imaging [43]. Even in clinically node-positive cases, the ability of axillary ultrasound and breast MRI to reliably distinguish the number of suspected nodes remains limited [44]. Consequently, prompt and precise detection of lymph node metastasis is essential for optimizing surgical strategies, radiation therapy planning, and treatment selection.

Lymph node metastasis can be predicted through either primary tumor or individual lymph nodes imaging, in which recent AI-based approaches have demonstrated promising performance. A deep-learning model based on ultrasound images of the primary tumor achieved an AUC of 0.90 in an internal test cohort and 0.89 in an external validation set [41]. Zheng et al. built a deep-learning radiomics model using breast ultrasound, shear-wave elastography, and clinical parameters to predict axillary lymph node status in early-stage breast cancer, achieving an AUC of around 0.90 for N0 vs. any metastasis and for low vs. heavy nodal burden in an independent test set [45]. Similarly, Nguyen et al. showed that preoperative dynamic contrast-enhanced MRI of the primary tumor alone could be used to predict clinical nodal status using a deep-learning framework, with the best-performing model achieving a sensitivity of 72% and an AUC of 0.71 [46]. Incorporating clinical information into the four-dimensional model resulted in additional improvements in AUC. Ren et al. reported that convolutional neural network (CNN) models applied to multiparametric MRI of individual lymph nodes yielded accuracies ranging from 86.08% to 88.50%, with AUC values between 0.804 and 0.882 [43].

In addition to local imaging features, other studies have reported strong performance using combined regions of interest and have directly compared deep-learning (DL) models with radiomics-based approaches. Gao et al. proposed a DL framework for axillary lymph node metastasis detection that integrates a three-dimensional deep residual network (ResNet) architecture with a convolutional block attention module, referred to as RCNet [47]. Their best-performing model achieved an AUC of 0.907, outperforming the corresponding radiomics-based support vector machine model, which attained an AUC of 0.856. These results are consistent with earlier studies that combined tumor and axillary lymph node radiomic features for nodal status assessment [48]. Similarly, Sun et al. reported that CNN-based models outperformed ultrasound-derived radiomics models in predicting axillary lymph node metastasis, with AUCs of 0.912 and 0.886, respectively [49]. In a prospective study, a CNN model trained on combined intratumoral and peritumoral regions achieved the highest AUC of 0.95 among all image-only models.

Whether clinical node-negative patients with image-detected nodal metastases are appropriate for sentinel lymph node biopsy or should proceed directly to axillary lymph node dissection or NAC remains controversial [32]. If lymph node metastasis criteria are redefined using AI-assisted imaging, new clinical trials may be required to validate their impact on clinical management.

## 3. AI for Treatment Response Prediction

In early-stage TNBC, neoadjuvant systemic therapy (NAST) has emerged as a cornerstone of clinical management. Administering therapy before surgery can shrink tumors and increase the likelihood of successful breast-conserving surgery [50]. This approach provides valuable prognostic information during treatment and allows adjustment of subsequent therapy based on response. As a result, NAST is now considered the standard of care for most TNBC patients, including early-stage cancers [51,52].

The backbone of NAST in TNBC has traditionally been multi-agent chemotherapy. The landmark NSABP B-18 trial established that delivering chemotherapy in the preoperative (neoadjuvant) setting achieves long-term survival rates equivalent to giving the same chemotherapy after surgery, while significantly increasing the likelihood of breast-conserving surgery [53,54]. More recently, the integration of immunotherapy into neoadjuvant regimens has further improved outcomes for early TNBC [55,56,57]. Pathologic complete response (pCR) after neoadjuvant chemotherapy is a particularly important endpoint in TNBC, as pCR is strongly associated with excellent long-term survival outcomes [58,59]. However, only about 50–60% of TNBC patients achieve a pCR, reflecting substantial heterogeneity in chemosensitivity [55]. Furthermore, the intensive treatments used in TNBC (multi-agent chemotherapy and especially immunotherapy) can carry substantial toxicities [55,60]. These side effects are generally acceptable if the therapy achieves a cure or major tumor response, but for patients who ultimately do not respond, the toxicity represents unnecessary harm without benefit. Therefore, the ability to predict which patients are likely to achieve pCR and which are likely to be non-responders before or early in treatment has become a crucial goal in TNBC management. Accurate response predictors would enable clinicians to tailor therapy accordingly.

Deep-learning neural networks have recently shown great promise in tackling the challenge of predicting therapy response in TNBC (Table 2). Unlike conventional radiologic assessment, DL methods can automatically learn complex, subtle imaging features from raw scans without manual feature selection. This capability is valuable for TNBC, where treatment response patterns can be too nuanced or multi-dimensional for human observers to discern. Deep-learning models, by detecting latent imaging patterns associated with chemo- or immunotherapy sensitivity, are well-suited to predict which patients are likely to achieve pCR versus those who will have residual disease.

**Figure 2:**
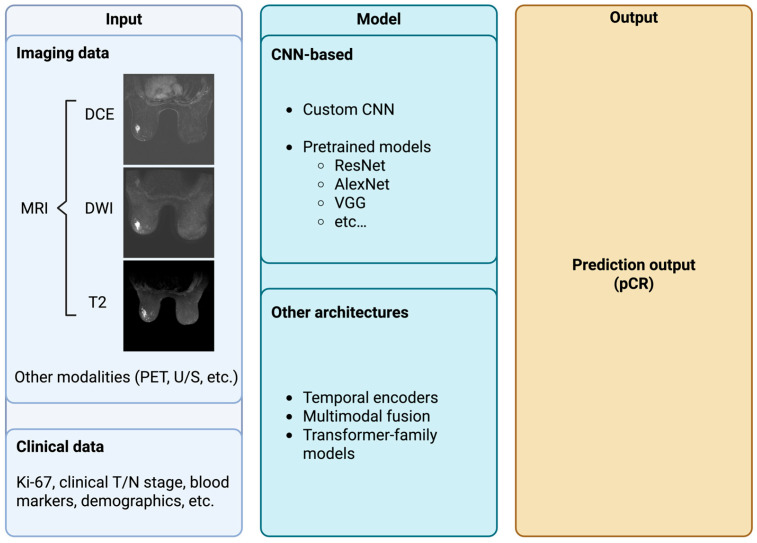
Summary of typical inputs and deep-learning architectures considered in pCR prediction models for breast cancer. (DCE = dynamic contrast enhanced MRI; DWI = diffusion weighted imaging; PET = positron emission tomography; U/S = ultrasound; CNN = convolutional neural network; VGG = visual geometry group; pCR = pathological complete response).

A typical MRI protocol for TNBC includes DCE-MRI to capture tumor vascularity and enhancement kinetics, diffusion-weighted imaging for tumor cellularity (via ADC maps), and T2-weighted sequences for morphology (assessing tumor shape or surrounding edema) [60]. Each sequence provides complementary cues about response: for example, the degree and pattern of contrast enhancement and functional tumor volume on DCE-MRI, as well as the pretreatment ADC values from DWI, have shown associations with pCR [70,71]. However, these single-sequence markers have only modest predictive power in isolation. This has motivated the integration of multiple MRI contrasts and time points into DL models to capture the full imaging phenotype of TNBC. This section reviews key considerations and developments in DL-based response prediction for TNBC, including the choice of input data modalities, use of longitudinal imaging, and network architectures. Typical inputs and deep-learning model architectures considered for pCR prediction in breast cancer are shown in Figure 2.

### 3.1. Data Inputs and Modalities

Early deep-learning studies have often explored single-image inputs, focusing on one MRI sequence. In 2019, Ha et al. trained a CNN model using only the pretreatment post-contrast T1-weighted MRI (a single phase in DCE-MRI) and achieved ~88% accuracy in a three-class prediction of complete vs. partial vs. no response [72]. While single-sequence models proved the feasibility of DL, they may miss complementary information available in other sequences. This has led to efforts combining multiple MRI contrasts with multiparametric models that incorporate multiple sequences in parallel. Joo et al. demonstrated that using both DCE subtraction images and T2-weighted images improved predictive performance compared to either alone, and further integrating clinical factors boosted the AUC to 0.888 [64].

In TNBC-specific cohorts, multiparametric approaches have also demonstrated some promising results. Zhou et al. developed a DL model incorporating both DCE-MRI and DWI scans as inputs and an AUC of ~0.86 on retrospective test data and ~0.83 on a prospective validation set of TNBC patients [66]. Interestingly, the authors found that not all MRI-derived inputs contributed equally. The network performed best when using the positive enhancement integral (PEI) map from DCE combined with a high b-value DWI, whereas including alternative DCE metrics (slope or signal enhancement ratio) or lower b-value/ADC maps can lead to poorer results [66]. This suggests that careful selection or derivation of input images can impact model performance.

Beyond MRI alone, researchers have explored other imaging modalities and data sources. Choi et al. applied a DL model to PET/MRI scans acquired early during NAST and showed that multimodality imaging could predict treatment response in advanced breast cancer with an AUC up to 0.805 [61]. In a TNBC-only study, Lyu et al. developed an ultrasound-based model that combines deep-learning, radiomics, clinical, and BI-RADS features to predict pCR, achieving an AUC of 0.84 in external testing [69]. Similarly, Massafra et al. achieved AUCs around 0.78–0.80 by merging MRI-based features with clinical data, outperforming imaging-only models [65]. These results underscore the feasibility and benefits of multimodal data fusion (whether across different imaging modalities or between imaging and non-imaging data) to enhance predictive accuracy.

### 3.2. Longitudinal Time Points

Many studies in this area incorporate longitudinal time points (early/mid/post-treatment in addition to baseline) to capture therapy-induced changes. These longitudinal models (especially the ones that incorporate post-therapy images after full cycles of NAST) tend to achieve better nominal performance than models that attempt to predict outcomes using only pretreatment baseline images. Krasniqi et al. found that adding at least one on-treatment acquisition significantly increased performance from a baseline-only median AUC of 0.82 to 0.91 [73]. From a clinical perspective, the greatest value comes from predictions using early or mid-treatment time points, which can enable actionable decision-making.

### 3.3. Model Architectures and Training Strategies

In addition, emerging work has begun to explore other advanced DL architectures, including vision transformers (ViT) and CNN–Transformer hybrids. Comes et al. developed an ensemble ViT model using pre- and mid-treatment DCE-MRI, achieving an AUC of 0.813 on external testing [67]. Song et al. also used a spatiotemporal ViT-based model on longitudinal DCE-MRI, achieving an AUC of 0.886 on external I-SPY2 testing data [74]. However, these approaches remain at an early stage, with limited evaluation in breast cancer- or TNBC-specific cohorts. Overall, CNNs remain the dominant architecture in pCR prediction studies. The trend is toward leveraging proven neural network designs and integrating novel and advanced architectural elements to improve performance or interpretability.

### 3.4. ROI Localization

Another consideration in deep-learning pipelines for treatment response is how the tumor region of interest (ROI) is defined and extracted from imaging. Historically, most studies have relied on manual tumor contouring by expert radiologists to precisely delineate the lesion on MRI scans. This ensures a highly accurate ROI, but it is labor-intensive, time-consuming, and subject to inter-observer variability. U-Net and its variants have become popular for breast tumor segmentation. A recent multi-institutional study demonstrated that a 3D U-Net could achieve radiologist-level tumor segmentation performance on breast DCE-MRI, with a median Dice score of 0.93 [75]. However, the performance varies across sites and requires harmonization to maintain robustness.

Given these trade-offs, some studies have explored simpler ROI-localization strategies, such as bounding boxes around the tumor in lieu of precise pixel-wise masks. Defining a lesion’s bounding box is generally faster and less subjective than detailed contouring. In practice, a coarse ROI (a bounding box around the enhancing tumor) might be obtained via a quick radiologist mark or a preliminary detection algorithm. The question is whether such an approach sacrifices prediction accuracy compared to exact segmentation. Xu et al. compared three ROI definitions (radiologist-drawn tumor contours, a tight bounding box, and an enlarged bounding box) and found that ROI choice had minimal impact on pCR prediction performance, with only a 0.03–0.04 difference in AUC across contouring approaches [76]. These results imply that while ROI localization remains necessary, labor-intensive manual segmentation may not be essential, and efficient semi-automated approaches could offer a practical balance between accuracy and clinical workflow integration.

## 4. AI for Prognosis and Risk Stratification

Beyond diagnosis and treatment response assessment, AI has increasingly been applied to prognostic modeling in TNBC to improve the prediction of long-term outcomes, including disease-free survival (DFS), recurrence-free survival (RFS), event-free survival (EFS), and overall survival (OS). Prognostic modeling addresses a different clinical problem than response prediction: rather than estimating sensitivity to a specific therapy, these models aim to characterize baseline (or early-course) disease aggressiveness and long-term risk. This distinction is particularly relevant in TNBC, where outcomes are heterogeneous and widely adopted genomic prognostic assays have not been established as routine tools for risk stratification as they are in some hormone receptor–positive settings.

Across the literature, AI-based prognostic approaches span a range of data types, from clinical variables alone to imaging-derived phenotypes and tissue-based molecular or histopathologic features (Table 3). In general, models that incorporate tumor-specific features may provide more refined stratification, but at the cost of increasing complexity and resource requirements. The sections below summarize this landscape, with emphasis on MRI-based AI as a practical middle ground between population-level clinical models and tissue-based assays.

### 4.1. Survival Prediction

Multiple studies support an association between pre-treatment MRI radiomic features and long-term outcomes in TNBC. Kim et al. extracted radiomic features from preoperative T2-weighted and contrast-enhanced MRI in 228 TNBC patients and derived a radiomics risk score using LASSO [77]. The score was associated with DFS in the training and validation cohorts and remained independent in a multivariable analysis adjusting for tumor size, nodal status, and histologic grade. Adding the radiomics score to clinicopathologic variables improved prognostic performance relative to clinicopathologic variables alone.

Zhao et al. developed a radiomics nomogram using preoperative dynamic contrast-enhanced MRI to predict RFS in 151 TNBC patients with external validation, reporting a concordance index of ~0.78 for the combined radiomics–clinical model [79]. High-risk patients identified by the radiomics signature experienced earlier recurrence, suggesting baseline MRI phenotypes capture aggressive disease biology beyond gross tumor burden.

Radiogenomic work further supports biological plausibility. Noor et al. trained a 20-feature MRI radiomics signature to identify a high-risk TNBC subgroup defined by a 50-gene transcriptomic profile [80]. Across independent cohorts, the imaging signature stratified patients into high- and low-risk groups with a ~25% absolute difference in 5-year OS and outperformed conventional clinicopathologic predictors. Collectively, these studies suggest baseline MRI may capture prognostically relevant information related to tumor heterogeneity, vascularity, and cellular organization.

AI has also been applied to large clinical datasets to provide population-level prognostic baselines. Xu et al. trained a deep neural network on SEER clinical variables (~37,800 TNBC patients), reporting C-indices of ~0.82 internally and ~0.76 on external testing, outperforming Cox models and AJCC staging [76]. In a complementary approach, Alzate-Granados and Niño clustered clinical data from >4800 TNBC patients into phenotypic subgroups with distinct relapse and survival profiles, then trained supervised models to predict 5-year outcomes (relapse AUC ~0.76) [81]. These approaches are scalable and rely on routinely available variables, but they are less tumor-specific than imaging, pathology, or molecular models.

AI-driven analysis of pathology and transcriptomic data provides biologically grounded prognostic signals. Zhang et al. introduced the TRIP system, a deep-learning framework applied to H&E whole-slide images across multicenter TNBC cohorts, reporting DFS C-indices of ~0.73–0.74 across internal and external datasets [83]. The authors also used heatmaps to highlight histologic regions contributing to risk stratification.

Across pathology AI studies, interpretability analyses often emphasize morphologic and microenvironmental features already known to be prognostically relevant in TNBC [83,84]. In particular, adverse outcomes have been associated with marked nuclear atypia, extensive necrosis, and low tumor-infiltrating lymphocytes (TILs), whereas lymphocyte-rich tumors tend to demonstrate more favorable long-term survival [83]. Independent pathology and translational studies similarly report that higher stromal and intratumoral TIL densities correlate with improved DFS and OS in TNBC [85,86].

Sandarenu et al. used multiple instance learning to predict breast cancer–specific survival in TNBC from histopathology, with an AI-derived risk score being independent on multivariable analysis (HR ~2.7 for cancer-specific death) [78]. At the molecular level, Kim et al. developed a 10-gene transcriptomic signature using machine learning to predict invasive DFS in early-stage TNBC, reporting strong stratification and independent prognostic value [87]. Pérez-Peña et al. likewise showed immune-related transcriptomic signatures associated with recurrence risk and survival in neoadjuvant-treated TNBC [88]. Together, these findings underscore the prognostic relevance of tumor immune contexture and cellular morphology and help anchor imaging-based and multimodal AI models in established TNBC biology.

### 4.2. Predicting Recurrence and Metastatic Risk

Beyond overall survival endpoints, AI models have begun to address recurrence risk and patterns of progression, extending prognostic modeling from whether disease recurs to how and when it recurs. This is clinically important in TNBC, where recurrences tend to occur earlier and are more frequently visceral.

MRI radiomics provides TNBC-specific evidence for recurrence stratification. Zhao et al. developed a DCE-MRI radiomics nomogram predicting RFS after surgery, with external validation (C-index ~0.78) and earlier recurrence among radiomics-defined high-risk patients [79]. Noor et al. similarly linked an MRI-derived radiomic signature to transcriptomic high-risk profiles and long-term outcomes, reinforcing imaging as a plausible noninvasive surrogate for molecular recurrence risk [80]. Longitudinal (delta) imaging features have also been associated with recurrence-free survival, although many studies include mixed breast cancer subtypes rather than TNBC alone [89].

In broader breast cancer cohorts, machine-learning models have demonstrated the feasibility of predicting distinct recurrence patterns. Kovács et al. reported models using clinical/pathologic variables to predict local recurrence localization and subsequent distant metastasis, with moderate discrimination (ROC AUC ~0.70–0.75) [90]. Shiner et al. similarly modeled site-specific distant metastasis risk (including visceral and brain involvement) with AUCs in a similar range [91]. Although these models were not restricted to TNBC, TNBC status emerged as a high-risk factor, consistent with its propensity for early visceral spread.

Despite these advances, TNBC-specific AI models for organ-specific metastatic prediction remain limited. Epidemiologic and clinical studies report elevated early brain and lung metastatic risk in TNBC relative to hormone receptor–positive disease, with central nervous system involvement often occurring within the first few years after diagnosis [46,92,93,94]. Most AI models to date emphasize overall recurrence/survival rather than explicitly modeling site-specific metastatic risk, likely reflecting smaller event counts per site, heterogeneous surveillance practices, and limited availability of large longitudinal datasets annotated for metastatic timing and organ involvement. As multimodal datasets mature, future work may enable more refined prediction of metastatic patterns and inform risk-adapted surveillance strategies in TNBC.

AI-based prognostic models may support risk-adapted management by identifying patients at increased risk of early recurrence despite standard therapy. MRI- and pathology-based approaches offer noninvasive or routinely available alternatives to molecular assays and may be useful when tissue is limited. Models integrating clinical, imaging, and molecular data appear most informative, but prospective validation and workflow integration remain necessary. At present, these tools should be treated as adjunct risk stratifiers that inform multidisciplinary decision-making.

## 5. Challenges and Future Directions

Despite substantial progress, several challenges remain before AI can be reliably integrated into the clinical management of TNBC. A fundamental limitation is the relative rarity and biological heterogeneity of TNBC itself. TNBC accounts for a minority of breast cancer cases. Many studies are based on relatively small or mixed-subtype datasets to achieve sufficient sample sizes. This can obscure TNBC-specific patterns and increase the risk that models learn dataset-dependent features rather than reproducible disease characteristics. Variability across histologic subgroups, treatment regimens, and clinical behavior further complicates model development and validation, all of which are unique challenges in TNBC. Addressing these challenges will require coordinated multicenter collaboration, harmonized imaging protocols, and data-sharing frameworks designed specifically for TNBC-focused research.

Another challenge concerns model performance and clinical sufficiency. Many AI models report strong discrimination metrics, with AUC values exceeding 0.85. However, these “best-performing” results are often derived from small retrospective datasets without robust external validation, which may reflect institution-specific biases, such as imaging characteristics, patient selection, or preprocessing, rather than a true biological signal. Nominally strong metrics may not generalize across patient populations, scanners, or treatment protocols. Furthermore, it remains unclear what level of performance is “good enough” for clinical decision-making. Incremental improvements in AUC may not translate into meaningful clinical benefit if sensitivity, specificity, or calibration are inadequate in real-world settings. Standard performance metrics do not fundamentally measure downstream clinical impact, such as changes in treatment selection, reduction in unnecessary toxicity, or improvements in survival. Future studies must therefore move beyond retrospective accuracy metrics and evaluate whether AI-assisted decisions improve patient outcomes through prospective and workflow-integrated studies.

Clinical interpretability represents another major barrier. AI is often valued for its ability to detect subtle imaging or pathological patterns that are imperceptible to human observers. At the same time, clinical adoption requires that AI outputs be understandable, explainable, and defensible to human experts. This creates an inherent paradox. Emerging approaches in explainable AI offer partial solutions by highlighting which inputs contribute most strongly to a given prediction. However, these explanations remain limited in the context of imaging data and are not yet standardized for clinical use. In practice, AI is likely to function best as a decision-support tool, augmenting rather than replacing clinician judgment, with outputs presented as risk estimates or probability scores accompanied by interpretable context.

Integration into clinical workflows poses additional challenges. Many AI pipelines rely on labor-intensive steps such as manual tumor segmentation, complex preprocessing, or offline analysis, limiting scalability. For routine clinical use, AI systems must be robust, automated, interoperable with existing PACS and electronic health record systems, and capable of operating across diverse scanners and institutions. Workflow studies and human–AI interaction research will be critical to determine how AI outputs should be displayed, interpreted, and acted upon by radiologists and oncologists without increasing operational burden.

Looking forward, several future directions may enhance the clinical relevance of AI in TNBC. Multimodal models that integrate imaging, pathology, clinical variables, and molecular data are likely to provide more comprehensive risk stratification than any single modality alone. Longitudinal modeling represents another promising avenue. Rather than generating static predictions at a single time point, AI systems may evolve toward dynamic frameworks that update predictions over the course of treatment, incorporating early response signals and clinical outcomes. Such “digital twin”–like approaches could enable adaptive treatment strategies, where model predictions inform decisions and subsequent outcomes are fed back to refine future predictions.

## 6. Conclusions

AI has demonstrated potential to augment multiple decision points in TNBC care, including improving diagnostic confidence in ambiguous imaging findings, identifying patients unlikely to benefit from standard neoadjuvant regimens, and refining post-treatment risk stratification to guide surveillance or adjuvant therapy selection. The clinical value of these systems will ultimately depend not on isolated performance metrics but on whether their use meaningfully alters management decisions and improves patient outcomes. Achieving this transition requires prospective validation in real clinical workflows, standardized reporting practices, and a clear definition of how model outputs should influence treatment pathways.

Future studies should focus on multicenter data sharing, consensus in imaging acquisition, and development of multimodal and longitudinal models capable of adapting to evolving disease states. Equally important are studies addressing human–AI interaction, model calibration across populations, and integration into existing clinical infrastructure. The central unmet need in AI for TNBC is not additional retrospective model development, but demonstration of reproducible benefit in routine care. With careful validation and clinically grounded implementation, AI can evolve from a predictive research tool into a practical component of personalized management for patients with TNBC.

## Figures and Tables

**Table 1:** Selected studies using AI for differentiation of TNBC from other subtypes of breast cancer.

Authors (Year)	MRI Modality	Total Patients	TNBC Patients, *n* (%)	Method	Performance
Leithner et al. (2020) [14]	DWI	91	23 (25)	Radiomics + ML	Acc: 73.4%
Ni et al. (2020) [15]	DWI	112	11 (10)	Radiomics + ML	Acc: 73.0%
Leithner et al. (2020) [9]	MP-MRI	91	23 (25)	Radiomics + ML	AUC: 0.86
Demircioglu et al. (2020) [16]	DCE-MRI	95	15 (16)	Radiomics + ML	AUC: 0.73
Wang et al. (2021) [17]	DWI	221	25 (11)	Radiomics + ML	AUC: 0.82
Zhang et al. (2021) [18]	DCE-MRI	99	10 (10)	Deep learning	AUC: 0.89
Huang et al. (2021) [11]	MP-MRI	162	25 (15)	Radiomics + ML	AUC: 0.965
Romeo et al. (2022) [13]	[18-F] FDG PET/MRI	98	25 (26)	Radiomics + ML	AUC: 0.887
Yin et al. (2023) [19]	MP-MRI	319	154 (48)	Deep learning	AUC: 0.94
Yue et al. (2023) [20]	DCE-MRI	516	72 (14)	Deep learning	AUC: 0.93
Zhang et al. (2023) [12]	MP-MRI	477	74 (16)	Radiomics + ML	AUC: 0.90
Huang et al. (2024) [10]	MP-MRI	188	33 (18)	Radiomics + ML	AUC: 0.86

**Table 2:** Selected studies using AI for treatment response prediction.

Authors (Year)	Input Data	Patient Cohort	Method	Performance
Ha et al. (2019) [61]	Pretreatment T1 + C	141 (mixed subtypes)	Custom 3D CNN	Acc = 88%
Choi et al. (2020) [62]	Single time point FDG-PET/CT, DWI	56 (mixed subtypes)	Custom 2D CNN (AlexNet)	AUC = 0.833
Qu et al. (2020) [63]	Longitudinal DCE-MRI, molecular subtype	302 (mixed subtypes)	Custom 3D CNN	AUC = 0.970
Joo et al. (2021) [64]	Pretreatment DCE-MRI, T2W, clinical factors	536 (mixed subtypes)	Pre-trained 3D ResNet50	AUC = 0.888
Massafra et al. (2022) [65]	Pretreatment DCE-MRI, clinical factors	225 (mixed subtypes)	Pre-trained 2D AlexNet	AUC = 0.803
Zhou et al. (2023) [66]	Longitudinal DCE-MRI, DWI	210 (TNBC only)	Custom 3D CNN	AUC = 0.86
Comes et al. (2024) [67]	Longitudinal DCE-MRI	106 (mixed subtypes)	Pre-trained 2D ViT-B-16	AUC = 0.813
Xu et al. (2025) [68]	Pretreatment DCE-MRI, DWI, clinical factors	344 (TNBC only)	Pre-trained 3D ResNets	AUC = 0.76
Lyu et al. (2025) [69]	Longitudinal Ultrasound, clinical factors	283 (TNBC only)	Custom fusion model (radiomics and MAE/ViT based)	AUC = 0.84

**Table 3:** Selected studies using AI for prognosis and risk stratification.

Authors (Year)	Input Data	Patient Cohort	Method	Prognostic/Outcome Variable	Performance
Kim et al. (2020) [77]	T2-weighted + DCE-MRI	228	Radiomics + ML	DFS	iAUC ≈ 0.77
Sandarenu et al. (2022) [78]	Histopathology	202	Multiple-instance learning	Cancer-specific survival	HR ≈ 2.7
Zhao et al. (2023) [79]	DCE-MRI	151	Radiomics + ML	RFS	C-index ≈ 0.78
Noor et al. (2025) [80]	DCE-MRI	749	Radiomics + ML	OS	AUC ≈ 0.71
Xu et al. (2025) [76]	Clinical variables	~37,800	Deep-learning survival model	OS	C-index ≈ 0.76 (external)
Alzate-Granados and Niño (2025) [81]	Clinical variables	4808	Unsupervised clustering + RF	Relapse risk; OS	AUC ≈ 0.76
Cheng et al. (2026) [82]	Ultrasound + multi-sequence MRI	103	Deep learning radiomics	DFS; OS	C-index ≈ 0.86 (DFS); 0.80 (OS)
Zhang et al. (2025) [83]	H&E whole-slide images	>450	Deep learning	DFS	C-index ≈ 0.73–0.74

## Data Availability

No new data were created or analyzed in this study. Data sharing is not applicable to this article.
